# Characterization of the Plant Growth-Promoting Activities of Endophytic Fungi Isolated from *Sophora flavescens*

**DOI:** 10.3390/microorganisms8050683

**Published:** 2020-05-07

**Authors:** Adiyadolgor Turbat, Dávid Rakk, Aruna Vigneshwari, Sándor Kocsubé, Huynh Thu, Ágnes Szepesi, László Bakacsy, Biljana D. Škrbić, Enkh-Amgalan Jigjiddorj, Csaba Vágvölgyi, András Szekeres

**Affiliations:** 1 Department of Microbiology, Faculty of Science and Informatics, University of Szeged, Közép fasor 52, H-6726 Szeged, Hungary; adiyadolgor_turbat@yahoo.com (A.T.); rakkdavid@gmail.com (D.R.); arunabio2011@gmail.com (A.V.); shigsanyi@gmail.com (S.K.); huynh_thu@hcmut.edu.vn (H.T.); mucor1959@gmail.com (C.V.); 2 Doctoral School in Biology, Faculty of Science and Informatics, University of Szeged, H-6726 Szeged, Hungary; 3 Department of Plant Biology, Faculty of Science and Informatics, University of Szeged, Közép fasor 52, H-6726 Szeged, Hungary; agnesszepesi79@gmail.com (Á.S.); bakacsy@gmail.com (L.B.); 4 Faculty of Technology, University of Novi Sad, Bulevar cara Lazara 1, 21000 Novi Sad, Serbia; biljana@tf.uns.ac.rs; 5 Laboratory of Microbiology, Institute of General and Experimental Biology, Mongolian Academy of Science, Ulaanbaatar 13330, Mongolia; Enkh27@yahoo.com

**Keywords:** endophytic fungi, plant growth-promoting activities, IAA (indole-acetic-acid) production

## Abstract

Endophytic fungi in symbiotic association with their host plant are well known to improve plant growth and reduce the adverse effects of both biotic and abiotic stresses. Therefore, fungal endophytes are beginning to receive increased attention in an effort to find growth-promoting strains that could be applied to enhance crop yield and quality. In our study, the plant growth-promoting activities of endophytic fungi isolated from various parts of *Sophora flavescens* (a medicinally important plant in Mongolia and China) have been revealed and investigated. Fungal isolates were identified using molecular taxonomical methods, while their plant growth-promoting abilities were evaluated in plate assays. Altogether, 15 strains were isolated, representing the genera *Alternaria, Didymella*, *Fusarium* and *Xylogone*. Five of the isolates possessed phosphate solubilization activities and twelve secreted siderophores, while all of them were able to produce indoleacetic acid (IAA) in the presence or absence of tryptophan. The endogenous and exogenous accumulation of IAA were also monitored in liquid cultures using the HPLC-MS/MS technique to refine the plate assay results. Furthermore, for the highest IAA producer fungi, the effects of their extracts were also examined in plant bioassays. In these tests, the primary root lengths of the model *Arabidopsis thaliana* were increased in several cases, while the biomasses were significantly lower than the control IAA treatment. Significant alterations have also been detected in the photosynthetic pigment (chlorophyll-a, -b and carotenoids) content due to the fungal extract treatments, but these changes did not show any specific trends.

## 1. Introduction

Endophytic fungi live in the tissues of various plants without causing any symptoms of disease in the host [1]. This ecological niche with the continual metabolic interactions between the endophytic microorganism and the host plant seem to serve a remarkable strong evolutionary pressure enhancing the synthesis of secondary metabolites of endophytes with novel properties [2]. Previous investigations of these microorganisms indicated that they are excellent producers of compounds that can be exploited for agrochemical or medicinal purposes due to their biological activity (e.g., antiviral, antimicrobial, anticancer, insecticidal, immunosuppressive and antioxidant effects) [3]. Furthermore, it has also been discovered that the produced compounds are occasionally the same as those produced by the respective hosts, which have been exclusively isolated from higher plants [4], including the cardiotonic digoxin (*Digitalis lanata*) [5], ginkgolides (*Ginkgo biloba*) [6], the antidepressant and antimicrobial hypericin (*Hypericum perforatum*) [7], the anticancer pro-drug podophyllo-toxin (*Juniperus communis*) [8], and the anticancer paclitaxel and its metabolites (*Taxus baccata*) [9]. 

*Sophora flavescens* (shrubby sophora) is an endangered medicinal plant species growing only in a small area in Mongolia. In traditional Chinese medicine, this herb is connected to numerous medicinal activities, such as antipyretic, anthelminitic, antimicrobial, insecticidal, anticancer, antiviral and diuretic properties [10]. The main chemical components of *S. flavescens* are alkaloid and flavonoid compounds [11,12]. Currently, there are only a few studies conducted on the fungal endophytes in *Sophora* species. In the work of Yao et al., 655 fungal strains representing 47 taxa were isolated from the root of *S. tonkinensis* and they were tested as potential biocontrol agents against the phytopathogens of *Panax notoginseng* [13]. Furthermore, endophytic fungi *Aspergillus terreus* and a *Penicillium* sp. were isolated from *S. flavescens* and their bioactive compounds were purified and identified [14,15]. According to Zhang et al., the *A. terreus* strain was also isolated from the seeds of this plant and the fungal production of a host metabolite was confirmed and optimized [16].

Host plants without endophyte-fungal association are devastated by the waves of extreme temperature, drought, salinity and pathogen attack [17,18,19,20]. Furthermore, endophytes are beneficial to the host plants in terms of the production of plant growth regulating hormones and the solubilization of minerals and their antagonistic behavior against plant pathogens and pests [21,22]. The mineral solubilization is the indirect way for the plant growth promotion, which could occur in the soil, when the nutritional elements will be consumable for plants, including nitrogen, phosphorus and mobilized metals [23,24,25]. As a direct way of plant growth promotion, it was demonstrated that endophytic fungi can produce phytohormones, especially gibberellins and indole acetic acid (IAA). Therefore, these endophytes, with their secreted plant growth regulating compounds, are of great potential interest to enhance crop yield and quality. These compounds can influence plant development as well as rescue plant growth in a stressful environment [26,27,28].

In this study, it was assumed that the fungal endophytes, living originally in the plant host, are able to influence the plant growth and fitness parameters of other plants through their promotion effects. Therefore, fifteen endophytic fungi were isolated from *S. flavescens* medicinal plant and both the indirect and direct plant growth-promoting activities of the identified strains were characterized including phosphate solubilization, siderophore- and IAA production. Furthermore, their possible growth-promoting effects were also evaluated in in vitro plant experiments monitoring plant fitness parameters.

## 2. Materials and Methods 

### 2.1. Isolation of Endophytes from Sophora flavescens

Plant specimens of *Sophora flavescens* were sampled from the territory of Dornod province in Mongolia (N49.148204, E114.875597), where, in winter, the average temperature is around from −27 to −32 °C with frequent snowfalls. In summer, the average temperature is 25–30 °C with cold nights of around 13 °C. The number of sunny days per year is 251–260 and the annual precipitation level averaged 276.9 mm. Each collected specimen (*n* = 4) was placed in a sealed plastic bag and labelled, numbered and noted with the date of collection and stored at 4 °C until processing. Plant samples subjected to a three-step surface sterilization procedure according to the method described by Vigneshwari et al. [7]. Portions of leaves, branches, roots and flowers were thoroughly washed in running tap water for 10 min to remove soil particles and adhered debris, then they were allowed to dry on the paper towel. The plant tissues were then cut into small pieces of less than 1 cm, which were washed in 70% ethanol for 1 min, in 50% sodium hypochlorite solution for 3 min and 70% ethanol for 30 s. After drying, each piece was cut to expose their inner tissue and placed on potato dextrose agar (PDA) or corn meal malt extract agar (CMM) supplemented with 50 mg/L chloramphenicol to suppress bacterial growth. All the plates were incubated at 25 °C for up to 7–10 days and were checked daily for the growth of fungal colonies. Pure fungal isolates were obtained by picking individual colonies from the PDA plates and plating on fresh PDA medium and incubating at 25 °C for 10 days. Each fungal culture was checked for purity and transferred separately to PDA slants and maintained at 4 °C as well as all isolates were deposited into the Szeged Microbiological Collection (SZMC, Hungary; http://szmc.hu/).

### 2.2. Identification of the Isolates

For DNA isolation, fungal isolates were grown in potato dextrose broth (PDB; VWR, Debrecen, Hungary) for 7 days at 25 °C. Isolation of genomic DNA from mycelia was performed using an E.Z.N.A. Fungal DNA Kit (VWR, Debrecen, Hungary) according to the manufacturer’s instructions. The internal transcribed spacer (ITS) region of the rDNA was amplified using the ITS1 (5’ TCCGTAGGTGAACCTGCGG-3’) and ITS4 (5’ TCCTCCGCTTATTGATATGC-3’) primers as described previously [29]. The sequencing of the amplified DNA fragments was performed on an ABI 373A DNA sequencer (Applied Biosystems Inc., Waltham, MA, USA) using dye dideoxy terminator reaction chemistry. The sequences were analyzed by BLAST similarity search at the website of the National Center for Biotechnology Information (http://www.ncbi.nlm.nih.gov/BLAST) based on their identity values (>99%). 

### 2.3. Phosphate Solubilization Assay

Cultures of endophytic fungi were grown on PDA medium for 7–10 days at 25 °C. The screening of fungal isolates for phosphate solubilizing activity was done on Pikovskaya’s (PKV) agar (5 g Ca_3_(PO_4_)_2_, 0.5 g (NH_4_)_2_SO_4_, 0.1 g MgSO_4_·7H_2_O, 0.2 g NaCl, 0.2 g KCl, 0.003 g FeSO_4_·7H_2_O, 0.003 g MnSO_4_·H_2_O, 10.0 g glucose, 0.5 g yeast extract, 15.0 g agar in 1 L distilled water) [30], and on modified Pikovskaya’s (MPKV) agar (5 g Ca_3_(PO_4_)_2_, 0.5 g (NH_4_)_2_SO_4_, 0.1 g MgSO_4_·7H_2_O, 0.2 g NaCl, 0.2 g KCl, 0.003 g FeSO_4_·7H_2_O, 0.003 g MnSO_4_·H_2_O, 2 g lecithin, 10.0 g glucose, 0.5 g yeast extract, 15.0 g agar in 1 L distilled water) [31,32]. After inoculation, the strains were grown for 48 h at 28 °C in three replicates, and, after the incubation period, the clear zones around the colonies were checked. 

### 2.4. Siderophore Production Assay

Siderophore detection was carried out according to the description of Schwyn and Nieland [33]. For this purpose, the isolated endophytes were precultured on PDA for 7–10 days at 25 °C. Agar plugs (3 mm in diameter) from the edges of young colonies were introduced to Blue Chrome Azurol A (CAS) agar medium. The plates were then incubated in the dark at 25 °C for 48 h. The color change of blue colored medium to yellow or orange indicates the positive result related the siderophore production.

### 2.5. IAA Production Assay

For this assay, the isolated endophytic fungi were precultured on PDA supplemented with tryptophan (Trp, 0.1% (*w/v*)) and were incubated at 25 °C for 7 days. Then, each strain was transferred with toothpicks aseptically onto the same medium with a 1.5-cm distance between each other, and the plate was overlaid with an 82-mm-diameter disk membrane Whatman 540 paper. After 24 h of incubation at 25 °C, reaching the colonies 0.5 to 2 mm in diameter, the membrane was removed from the plate and saturated with Salkowski reagent by immersing directly in petri dishes filled with the reagent. The tests were carried out at room temperature separately with two different formulations of the reagents (Reagent A: 2% 0.5 M FeCl_3_ in 35% HClO_4_; Reagent B: 1.2% FeCl_3_ in 37% H_2_SO_4_.) with three replicates. The reaction was allowed to proceed until adequate color developed and fungi producing IAA were identified by the formation of a characteristic red halo within the membrane surrounding the colony [34].

### 2.6. HPLC-MS/MS Measurement of IAA Production

The isolates were cultured in three replicates for 7 days at 25 °C in 30 mL of potato dextrose broth (PDB) in the presence (0.1 g/L) and absence of Trp on an orbital shaker at 200 rpm. Then, the extraction was carried out according to Sujit Shah et al. with minor modifications [35]. The mycelia were separated from the broth by filtration through a cheesecloth. Then, the oven-dried mycelia were extracted three times with 15 mL ethyl acetate, while the ferment broth (20 mL) was extracted sequentially three times with 20 mL ethyl acetate. The organic solvents from both pooled extracts were removed by a rotary evaporator (IKA HB10 basic, VWR, Debrecen, Hungary) in vacuum at 30 °C. The resulting four dry samples per each isolate (mycelia and broth, with and without Trp) were stored at −20 °C and resuspended in 1 mL of HPLC grade methanol prior to use. 

The analytical measurements were conducted on a Nexera XR HPLC system (Shimadzu Corporation, Kyoto, Japan) composed of a quaternary pump (LC-20ADXR), an auto sampler (SIL-20AXR), a column oven (CTO-10-ASVP) and a degasser (DGU-20A5R) coupled to a TSQ Quantum Access (Thermo Fischer Scientific, Waltham, MA, USA) triple quadrupole mass spectrometer (MS/MS). Chromatographic separations of samples (5 µL) were performed at 40 °C using a Phenomenex Gemini NX C18 50 mm × 2 mm, 3 µm column (Gen-Lab, Budapest, Hungary), coupled with a guard column with the same stationary phase using gradient elution. Eluent A was H_2_O and eluent B was acetonitrile supplemented each with 0.1% formic acid. The mobile phase flow rate was 0.3 mL/min and the gradient program started with eluent B at 20% for 1 min changing to 27% until 3.04 min, which was increased to 95% until 3.2 min with the value being held for five minutes then decreased to the initial 20% in 0.2 min and kept constant for 4 min, resulting in a run of 11.4 min in total. The MS ion source operated in positive electrospray ionization (ESI) ionization with the following ion source parameters: Spray Voltage, 4000 V; Vaporizer Temperature, 379 °C; Sheath Gas Pressure, 20; Sweep Gas Pressure, 2, Aux Gas Pressure, 55. The Capillary Temperature, Capillary Offset and Tube Lens Offset parameters were 250, 35 and 70 °C, respectively. The analyzers worked in multiple reaction monitoring (MRM) mode with a 0.015-s Scan Time and 2.4 mTorr Collision Pressure applying the *m/z* 176->130 and *m/z* 176->103 transitions for the IAA at Collision Energies of 13 and 31 V, respectively. 

The instrument control, data acquisition and evaluations were carried out with Xcalibur 1.7 (Thermo Fischer Scientific, Waltham, MA, USA) and Trace Finder 2.6 (Thermo Fischer Scientific, Waltham, MA, USA) software.

For the quantitative determination, the standard stock solution (1 mg/mL) of IAA (Sigma-Aldrich, Budapest, Hungary) was prepared in methanol. A series of calibration levels (*n* = 7) ranging in concentration from 0.05 to 5 µg/mL was prepared by appropriate dilution of the stock solution with methanol. 

### 2.7. Bioactivity Test of Extracts on Arabidopsis thaliana

To study the effect of the extract of IAA producing endophytic fungi isolates on plant growth, *A. thaliana* (Col-0 ecotype) seeds were planted on 0.5 × Murashige and Skoog agar (MS) medium (0.8%) [36] with the addition of 0.5% sucrose (*w/v*) (pH adjusted to 5.5 with NaOH) in plastic vertical Petri dishes (90 mm-diameter × 17 mm-high), five seeds per Petri dish in one line. The experimental setup was structured as described by Marik et al. [37]. Briefly, seeds were surface sterilized with 70% ethanol for 1 min, treated with 4% hypochlorite for 15 min and washed with sterile distilled water. After vernalization at 4 °C for 24 h, the seeds were sown onto the agar plates. The plants were then placed in a greenhouse with a photoperiod of 12 h of light and 12 h of darkness, a light intensity of 300 μmol/m^2^/s and a temperature of 25 ± 1 °C. After the three days of germination, plates were placed at an angle of 50° to allow the plants to develop and 5-mm holes were bored with a sterile cork borer 0.5 cm away from the root tips of 5-day-old *Arabidopsis* seedlings and filled with 50 μL of methanolic solutions of mycelial and broth extracts. For the assays, the IAA were tested in five concentrations (100, 10, 1, 0.1 and 0.01 µg/mL) and the ferment broth extracts were diluted also to same concentration levels based on their original IAA contents. For the controls, the untreated plants and methanol (50 μL) were used. Primary root growth was measured every 24 h for 4 days. The fresh weights of the plants from each plate were measured, and photosynthetic pigments were quantified, as described by Faragó et al. [38]. 

### 2.8. Statistical Analysis

All the statistical analyses were performed using GraphPad Prism version 7.0 for Windows (GraphPad Software, San Diego, CA, USA, 2016). In the bioactivity tests, the significant differences of the plant fitness parameters were determined by one-way analysis of variance with Bonferroni’s multiple comparison tests.

## 3. Results

### 3.1. Isolation and Identification of Endophytic Fungi

For the isolation, the host plant *S. flavescens* was collected from a specific location in the northeastern part of Mongolia. After the sampling, the leaf, stem, root and flower parts were separated, and these parts were examined for their fungal endophyte content. Altogether, sixty plant parts (15 parts from leaf, 15 from stem, 15 from root, 15 from flower) were applied for the isolation, from which fifteen endophytes were isolated in pure form from the leaves (4), stems (6) and roots (5). The molecular identification of the fungal endophytes was carried out by the comparative sequence analyses of the standard fungal sequence marker ITS rDNA. Based on the similarity to Blastn hits of the NCBI database, we were able to identify one isolate at the species level and 14 strains at the genus level. The isolates belonged to four genera involving *Alternaria* (3), *Didymella* (5), *Fusarium* (6) and *Xylogone* (1), and the accession numbers to the GenBank database are listed in Table 1.

### 3.2. Plant Growth-Promoting Traits of the Endophytes

**Figure 1:**
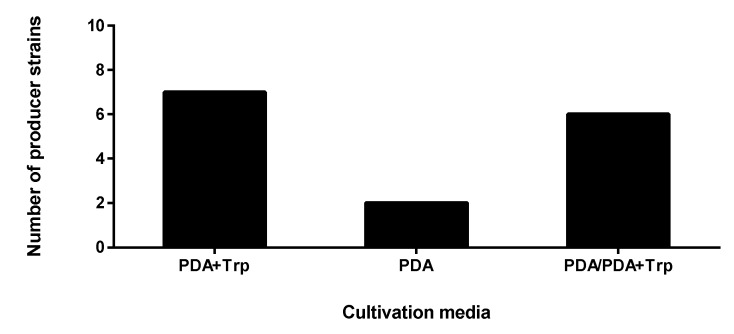
Indoleacetic acid (IAA) activities of endophytic fungi isolated from *S. flavescens* by agar plate assay. The PDA+Trp group produced IAA only in the presence of Trp; the potato dextrose agar (PDA) group produced IAA only without Trp; the PDA/PDA+Trp group showed IAA production in both cultivation conditions.

To test the plant promoting activity of the isolates, three different assays were applied. The assay of the plant hormone secretion revealed the direct stimulating activity of the endophytes, while examination of phosphate mobilization and siderophore production characterized indirect plant promoting traits. In the case of screening for plant hormone production, all fifteen strains proved to produce IAA either in the presence or in the absence of Trp, but certain isolates presented IAA in both cultivation conditions (Figure 1). There was no difference between the results of the perchloric and sulfuric acid containing reactions. Both reagents develop the same colour depending on the IAA concentration; however, when the perchloric acid is substituted for sulphuric acid, the sensitivity could be improved [39]. 

The highest number of isolates were able to produce IAA in the Trp-supplemented media involving the following isolates: two *Didymella* sp. (SZMC 26648 and 26650), three *Fusarium* sp. (SZMC 26660, 26654 and 26657), one *Alternaria* sp. (SZMC 26652) and one *Xylogone*. The following strains showed IAA production on both the Trp- and non-Trp-containing media, while the two *Fusarium* sp. (SZMC 26656 and 26658) endophytes presented positive IAA results only on the media without Trp supplementation: three *Didymella* sp. (SZMC 26647, 26649 and 26655), two *Alternaria* sp. (SZMC 26651, 26653) and one *Fusarium* sp. (SZMC 26659).

**Figure 2:**
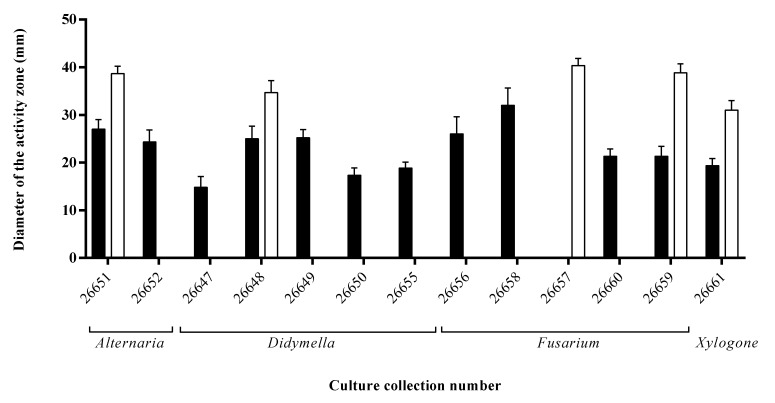
Phosphate solubilization (white bars) and siderophore production (black bars) of *S. flavescens* endophytes examined by agar plate assays.

During the siderophore detection assay, twelve endophytes caused orange/yellow zones around their colonies on CAS agar plates as a result of their siderophores sequestering and binding iron from the medium (Figure 2). The largest zone appeared on the plate of the *Fusarium* sp. SZMC 26658 strain, but also high siderophore productions were detected at the *Fusarium* sp. SZMC 26656 and at the *Alternaria* sp. SZMC 26651.

Regarding the third examined plant growth-promoting activity, one third of the isolates were capable of solubilizing phosphate when tested on PKV agar medium. The highest activity was detected in the case of *Fusarium* sp. SZMC 26657 strain (Figure 2).

### 3.3. IAA Production Confirmation of the Endophytes

As IAA has direct influence on plant growth, this activity of the isolates was confirmed via HPLC-MS/MS analyses using MRM mode. The detection limit of the developed method was 5 µg/mg and 5 µg/mL for the mycelia and the ferment broth, respectively. In contrast to the plate assay, IAA production could be measured more specifically, because the mycelia and the ferment broth could be examined separately for the hormone content. 

**Figure 3:**
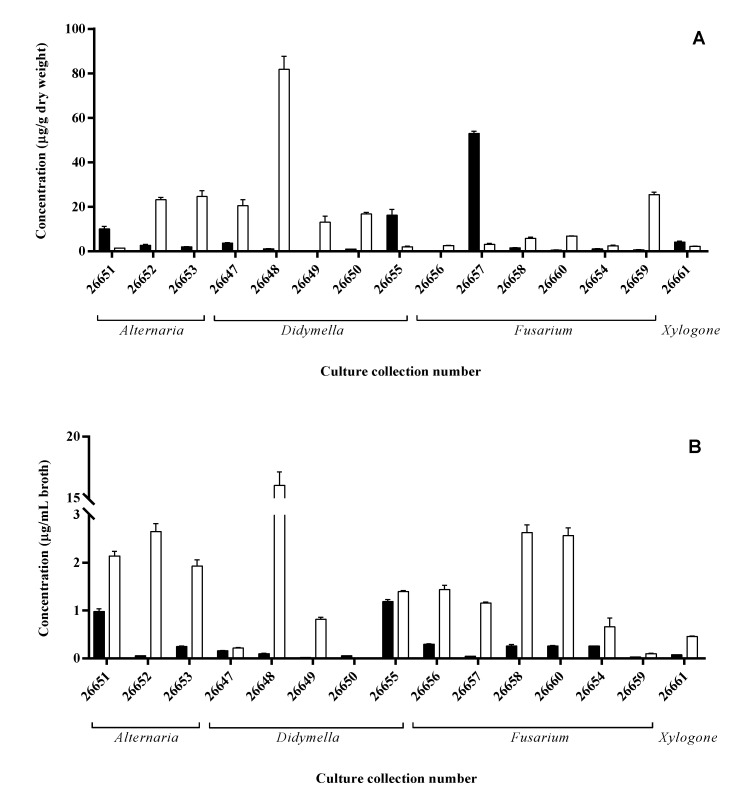
IAA content of mycelia (**A**) and ferment broth (**B**) of *S. flavescens* endophytes cultivated in PDB media (black bars) or PDB supplemented with Trp (white bars) measured by HPLC-MS/MS.

Similar to the results of the plate assays, each isolate showed remarkable IAA production either via the Trp-dependent or Trp-independent pathway (Figure 3). In the case of mycelial extracts, three strains accumulated a remarkable amount (>10 µg/g dry weight) of IAA in the PDB media including a *Fusarium* (SZMC 26657), a *Didymella* (SZMC 26655) and an *Alternaria* (SZMC 26651) isolate, while seven strains proved to be an outstanding producer on the media supplemented with additional Trp source reaching up to 82 µg/g dry weight at the *Didymella* (SZMC 26648) isolate (Figure 3A). 

Extracts of two isolates (SZMC 26655, 26651) contained high IAA amounts in their mycelia and presented IAA in the ferment broth in a remarkable concentration (>1 µg/mL broth) on simple PDB media. It seems that these strains accumulate and secrete the synthetized IAA, while the SZMC 26657 strain was not able to release the produced hormone into the cultivation media. In Trp-supplemented media, the highest IAA production was detected also for SZMC 26648, but significant productions were measured in case of SZMC 26652, 26653 (*Alternaria* sp.) and SZMC 26649 (*Didymella* sp.), similar to the mycelial results. However, in contrast to the mycelial IAA levels, decreased productions were observed for SZMC 26647, 26650 and 26659, while increases were found at eight isolates (Figure 3B). Based on the results, it could be concluded that the IAA productions were generally improved due to the additional Trp in the ferment broth and mainly in the mycelia except for SZMC26651, 26655 and 26657.

### 3.4. Bioactivities of Endophyte Extracts on Arabidopsis thaliana Plants

In order to evaluate the effects of the endophyte IAA on the plants, the ferment broth extracts of six selected isolates, showing the highest IAA productions in the presence of Trp, were investigated for growth-promoting activities in *A*. *thaliana*. Extracts were diluted to 100, 10, 1, 0.1 and 0.01 µg/mL based on their original IAA contents, and their impacts were compared both to the standard IAA solutions and to the pure solvent (methanol) as well as to the untreated plants. 

**Figure 4:**
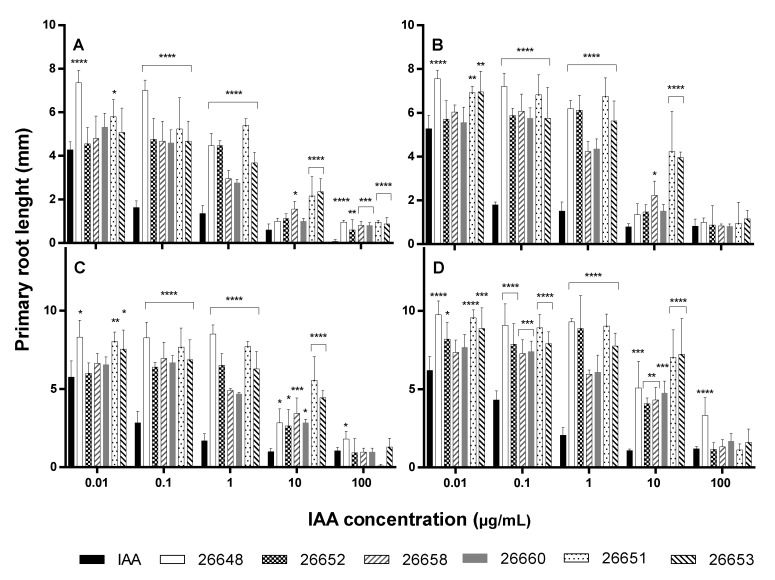
Primary root growth of 6- (**A**), 7- (**B**), 8- (**C**), and 9-day-old (**D**) *A. thaliana* plants after treatment with standard solutions and endophyte extracts at different IAA concentration levels. Significance is assessed based on *p*-values: * *p* ≤ 0.05; ** *p* ≤ 0.01; *** *p* ≤ 0.001 and **** *p* ≤ 0.0001.

Monitoring the lengths of primary roots, the solvent only slightly inhibited the root growth compared to the untreated control. In the experiment, the roots of control plants showed 2.16 ± 0.46 mm, 3.12 ± 0.69 mm, 3.56 ± 0.46 mm and 4.08 ± 0.58 mm growth, while the methanol-treated plants developed their roots up to 0.96 ± 0.22 mm, 1.68 ± 0.46 mm, 2.40 ± 0.35 mm and 2.80 ± 0.40 mm on the 6th, 7th, 8th and 9th days, respectively. The activities of the methanolic ferment broth extracts were compared to the IAA solutions, which were diluted with the same organic solvent (Figure 4). 

The exogenous IAA influenced the root elongation with a dose dependent manner according to the literature [40]. At the higher concentrations, the growth of primary roots were inhibited (1 µg/mL, 10 µg/mL and 100 µg/mL), while it was stimulated at the lower levels of IAA (0.01 µg/mL and 0.1 µg/mL) compared to the methanolic control experiments. However, the lengths of all *Arabidopsis* roots treated with fungal IAA were significantly higher than those of the IAA treatments at concentrations of 0.1 and 1 µg/mL at all days (Figure 4). Therefore, the fungal extracts could cause higher growth promotion than the pure IAA solutions containing the same levels of plant hormone. Furthermore, in the case of extracts of the SZMC 26651, 26653 (*Alternaria* sp.) and SZMC 26658 (*Fusarium* sp.) strains, these promoting effects remained at the 10 µg/mL level with high significances at all days. At the 100 µg/mL concentration level, remarkable differences were observed only at the 6th day between the effects of the IAA solution and the fungal extracts on the primary root growth, but later these disparities disappeared (Figure 4). 

**Figure 5:**
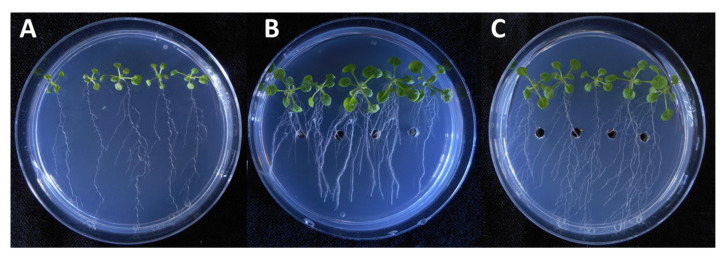
Effects of a selected fungal extract on the growth of *Arabidopsis* seedlings. Untreated control (**A**), 0.01 µL/mL of IAA standard (**B**) and the extract of SZMC 26648 broth containing 0.01 µL/mL of IAA (**C**).

It is also important to consider that, on the 9th day of incubation, the fungal extract more efficiently promoted the primary root growth than the standard solution almost at the all applied concentration levels. In the experiment, both the pure IAA and the fungal extract treatments lead to a substantial increase in root proliferation and lateral root growth. However, the application of the fungal extract in the plant assay showed that different types of growth developed longer and thinner root branches (Figure 5). 

**Figure 6:**
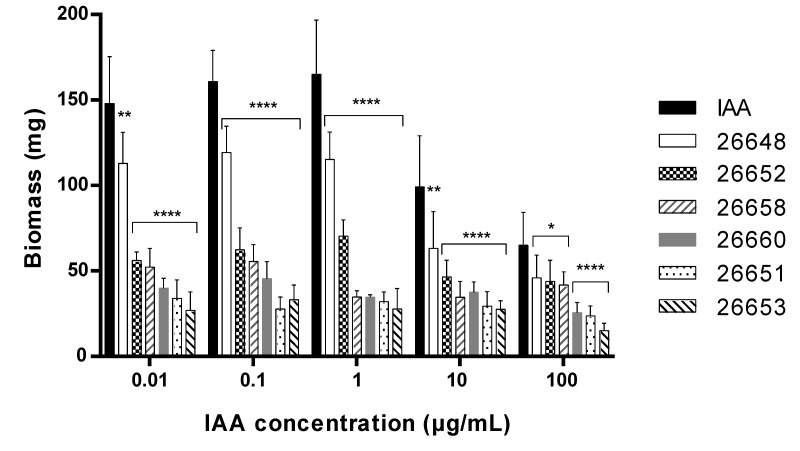
Biomass of 15-day-old *Arabidopsis thaliana* plants after treatment with the ferment broth extracts of selected endophytes diluted to five concentration levels based on their IAA content. The IAA standard was used for the control plants. Significance is assessed based on *p*-values: * *p* ≤ 0.05; ** *p* ≤ 0.01; *** *p* ≤ 0.001 and **** *p* ≤ 0.0001.

Comparing the biomass measured in the plant assays, the IAA treatment lead to a remarkably higher level of biomass; this was observed in plants treated with fungal extracts at each concentration level (Figure 6). In the case of the untreated and the methanolic control experiments, the biomasses were 0.03 ± 0.01 mg and 0.02 ± 0.01 mg, respectively. These values were comparable to the biomass of plants treated with the extracts of SZMC 26651, 26653, 26658 and 26660, but were remarkably lower than the biomass production of SZMC 26652 (*Alternaria* sp.) and 26648 (*Didymella* sp.). In the last two cases and for the pure IAA, the dose dependence of the biomass production was observed, namely increased productions were measured at lower treatment concentrations, while production plateaued in the concentration range of 0.01–1 µg/mL (Figure 6).

**Figure 7:**
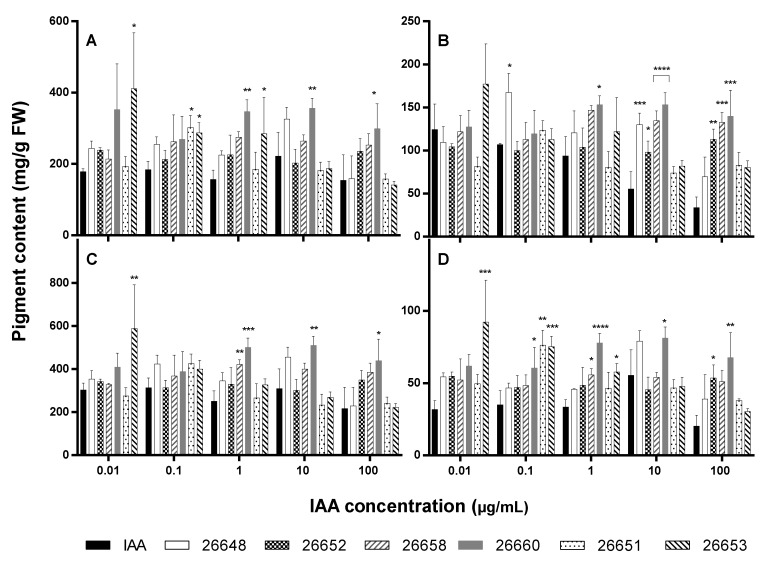
Pigment content of 15-day-old *A. thaliana* plants after treatment with ferment broth extracts of selected endophytes diluted to five concentration levels based on their IAA content: chlorophyll-a (**A**), chlorophyll-b (**B**), total chlorophylls (**C**) and carotenoids (**D**). IAA standard was used for the control plants. Significance is assessed based on *p*-values: * *p* ≤ 0.05; ** *p* ≤ 0.01; *** *p* ≤ 0.001 and **** *p* ≤ 0.0001.

Chlorophyll-a, -b and carotenoid levels were also altered significantly in certain cases after treatment with extracts compared to the IAA solutions (Figure 7).

Treatment with a fungal extract at 0.01 µg/mL IAA level resulted in a similar rate of production of photosynthetic pigments than with the IAA solution except for SZMC 26653 possessing an increased amount of chlorophyll-a and total chlorophyll as well as carotenoids. The leaves of plants treated with the extracts of strains SZMC 26651, 26653 (at 0.1 µg/mL); SZMC 26660, 26653 (at 1 µg/mL); and SZMC 26660 (at 10 µg/mL and 100 µg/mL) contained higher chlorophyll-a, while SZMC 26648 (at 0.1 µg/mL); SZMC 26660 (at 1 µg/mL); SZMC 26648, 26652, 26658 and 26660 (at 10 µg/mL) as well as SZMC 26652, 26658 and 26660 (at 10 µg/mL) comprised increased quantities of chlorophyll-b pigment than the pure IAA treated plants. In case of carotenoids, the extracts of SZMC 26660, 26651, 26653 (at 0.1 µg/mL); SZMC 26658, 26660, 26653 (at 1 µg/mL); SZMC 26660 (at 10 µg/mL) and SZMC 26652, 26660 (at 100 µg/mL) induced the production in the plants. Pigment contents for control and methanol-treated plants were the following: chlorophyll-a, 250.77 mg/g FW and 446.35 mg/g FW; chlorophyll-b, 114.79 mg/g FW and 207.32 mg/g FW; total chlorophylls, 365.55 mg/g FW and 654.67 mg/g FW; carotenoids, 58.79 mg/g FW and 103.22 mg/g FW, respectively.

It seems that the collected data did not follow a specified trend, but it could be highlighted that all pigments were found in increased amounts in the plants treated with the extract of SZMC 26660 at 1, 10 and 100 µg/mL IAA concentrations.

## 4. Discussion

In this study, for the first time, the plant growth-promoting activities of endophytes isolated from various organs of *S. flavescens* (shrubby sophora) were analyzed in order to compare and evaluate their effects on root growth, biomass and the photosynthetic pigments (chlorophyll-a, -b and carotenoids) in *A. thaliana*. Previous studies on fungal endophytes inhabiting the species *S. flavescens* comprised only two fungal genera, *Aspergillus* and *Penicillium* [14,15,16]. Based on our examinations, the fungal isolates represented the genera of *Alternaria*, *Didymella*, *Fusarium* and *Xylogone*, which have not been previously reported as fungal endophytes of *S. flavescens*. Furthermore, indirect plant promoting activities of the isolates were examined, including phosphate mobilization and siderophore production. It could be concluded that siderophore production was more common within the isolated fungal endophytes than phosphate solubilization. From the 15 strains, almost all produced siderophores in a measurable amount except for the *Fusarium* sp. SZMC26657 strain, while only five showed phosphate mobilization activities. Regarding the IAA plate assay, each isolate proved to produce IAA, from which seven strains showed production only in the presence of Trp and two only in the absence of Trp as well as six in both cultivation conditions. It is important to emphasize that the four strains SZMC 26659 (*Fusarium* sp.), 26661 (*Xylogone sphaerospora*), 26651 (*Alternaria* sp.) and 26648 (*Didymella* sp.) were positive for those three plant growth-promoting assays. These strains were isolated from different plant organs, including stem (SZMC 26651, 26659), leaf (SZMC 26658) and root (SZMC 26661). Based on these results, it seems that the plant growth-promoting activities are independent from the isolation source (plant part) and the taxonomical position of the isolates.

To further confirm the fungal production of IAA, samples were subjected to HPLC-MS/MS analysis, in which pure IAA was used as standard. In this experiment, certain extracts that originated from ferment broth or the mycelia of the examined strains cultured in the presence or absence of Trp contained IAA. Comparing the IAA production results of the plate assay and the HPLC-MS/MS measurements, in several cases, the gathered data did not correspond exactly with each other, which were also found in the case of endophytic bacteria reported in the literature [41]. Thus, it could be concluded that the plate assay is applicable rather as a pre-screening for the indication of the IAA production than the exact determinations due to the possibility of false positive results.

A growing number of studies are reporting the IAA production of endophytic fungi. Waqas et al. isolated 18 endophytes from roots of field-grown cucumber plants, from which two strains produced varying levels of IAA in their culture filtrate. The range of IAA production with or without Trp was found to be 3.89 μg/mL in *Phoma glomerata*, while *Penicillium* sp. produced a significantly higher amount of IAA (29.8 μg/mL) [42]. In the report of Khan et al. only a *Aureobasidium* sp. showed potential for IAA production in the L-tryptophan-independent pathway from 17 endophytes isolated from *Boswellia sacra*, an economically important frankincense-producing tree, while, in the L-tryptophan-dependent pathways, almost all the fungal strains showed the potential to produce IAA. In this case, the *Aureobasidium* sp. showed the highest potential for IAA production, which was 544.8 ng/mL of IAA [43]. Later, the IAA production of a *Preussia* sp. isolated in that study was also determined, where the production was 1.64 μg/mL based on GC MS/SIM analysis [44]. Khan et al. also published the IAA production of a *Paecilomyces formosus* endophyte isolated from the roots of cucumber plant, where the quantity of IAA was 10.2 μg/mL [27]. An *Aspergillus japonicus* endophyte strain, isolated from the wild plant of *Euphorbia indica* was also able to produce IAA at concentrations of 19.19 μg/mL, which facilitated the host plant growth under both normal and heat stress conditions [45]. From arabica coffee, twenty-seven endophytic fungal isolates were obtained and only one isolate of the *Colletotrichum fructicola* strain displayed positive IAA production when tested by the colorimetric assay of Salkowski’s reagent. This IAA production was confirmed via HPLC analysis and the IAA was determined at the level of 1205.58 μg/mL in optimal conditions at 26 days after cultivation [46].

In summary, previous reports have shown that the in vitro IAA level produced by endophytic fungi ranged from 0.5 to 1205.6 μg/mL, while, in our study, the IAA production of the isolated endophytes in the ferment broth ranged from 0.02 to 1.2 μg/mL and from 0.1 to 16.0 μg/mL, in the absence and presence of Trp, respectively, with the highest amount of IAA produced by *Didymella* sp. SZMC 26648 strain. However, in our cases, the cultivation conditions were not optimized. Furthermore, it is important to consider that the literature did not contain data about the IAA contents of the fungal mycelia, which were ranged in our experiments from 0.4 to 52.9 μg/mL and from 1.5 to 81.8 μg/g in dry weight, in the absence and presence of Trp, respectively, which were higher than the ferment broth IAA content.

*A. thaliana* has become a recognized model to analyze non-mycorrhizal plant-microbe interactions [47]. According to the Dovana et al., the root system extension of this organism could be changed considerably and significantly in plant–fungal co-cultures, including both decreasing and increasing effects. Furthermore, the presence of fungal endophyte also affects root architecture of *A. thaliana* such as the number of lateral roots and the root area [47]. The production of hormones by fungal endophytes could play an essential role in the growth and development of the host plant. In the study of Khan et al., the application of IAA-producing endophytes on the host plant appreciably increased shoot length, leaf number, internodes and quantities of photosynthetic pigments (chlorophyll a, b and total carotenoids) [43]. This is in agreement with previous reports, which showed that endophytic inoculation to the host plants resulted in the improvement of growth and stress tolerance [26,27,28]. However, the elongation of rice, corn and rye coleoptile segments induced by the crude IAA extract of *C. fructicola* endophyte were not statistically different from those of the pure IAA treatments, but the values were significantly higher than the control (distilled water) treatment [46].

In our work, the crude fungal IAA of the isolated endophytes at concentrations of 0.1 and 1 µg/mL promoted the elongation of the lengths of all *Arabidopsis* roots significantly, but these effects were not unequivocal at the higher IAA concentrations. Similarly, IAA production by endophytic fungi including *Preussia* sp., *Aspergillus japonicus* and *F. oxysporum* stimulated rice and corn root growth [44,46,48]. Interestingly, the biomasses of *Arabidopsis* plants were reduced due to the treatment of the fungal extracts, while the accumulation of photosynthetic pigments were increased only in certain cases.

Besides their longstanding importance in traditional health care, medicinal plants offer a unique source to gain bioactive endophytes, which could be used for various other applications. Phosphate mobilization as well as the phytohormone and siderophore production of certain fungal endophytes could be potentially important for agriculture/horticulture if they could be integrated in cultivation technologies of economically important crop plants. Our work provided novel information to this field and contributed to a better understanding of the plant growth-promoting phenomena of fungal endophytes. However, the effects of these parameters are rather complex, which need to be further analyzed using multifaceted approaches, especially if applications in field conditions will be considered.

## Figures and Tables

**Table 1:** List of the isolated and identified endophytes of *S. flavescens*.

Fungal Species	Culture Collection ID	Plant Part	Genbank Accession Number
			ITS
*Alternaria* sp.	SZMC 26651	Plant 1, Stem	MT218406
*Alternaria* sp.	SZMC 26652	Plant 2, Leaf	MT218412
*Alternaria* sp.	SZMC 26653	Plant 4, Leaf	MT218411
*Didymella* sp.	SZMC 26647	Plant 1, Leaf	MT218409
*Didymella* sp.	SZMC 26648	Plant 3, Leaf	MT218408
*Didymella* sp.	SZMC 26649	Plant 1, Root	MT218413
*Didymella* sp.	SZMC 26650	Plant 2, Stem	MT218407
*Didymella* sp.	SZMC 26655	Plant 4, Root	MT218401
*Fusarium* sp.	SZMC 26656	Plant 3, Root	MT218405
*Fusarium* sp.	SZMC 26657	Plant 3, Stem	MT218404
*Fusarium* sp.	SZMC 26658	Plant 2, Stem	MT218403
*Fusarium* sp.	SZMC 26660	Plant 4, Stem	MT218399
*Fusarium* sp.	SZMC 26654	Plant 4, Root	MT218410
*Fusarium* sp.	SZMC 26659	Plant 2, Stem	MT218402
*Xylogone sphaerospora*	SZMC 26661	Plant 3, Root	MT218400

## References

[1] Gouda S., Das G., Sen S.K., Shin H.-S., Patra J.K. (2016). Endophytes: A Treasure House of Bioactive Compounds of Medicinal Importance. Front. Microbiol..

[2] Schulz B., Boyle C., Draeger S., Römmert A.-K., Krohn K. (2002). Endophytic fungi: A source of novel biologically active secondary metabolites. Mycol. Res..

[3] Strobel G., Daisy B. (2003). Bioprospecting for microbial endophytes and their natural products. Microbiol. Mol. Biol. Rev..

[4] Kusari S., Spiteller M., Roessner U. (2012). Metabolomics of endophytic fungi producing associated plant secondary metabolites: Progress, challenges and opportunities. Metabolomics.

[5] Kaul S., Ahmed M., Zargar K., Sharma P., Dhar M.K. (2013). Prospecting endophytic fungal assemblage of *Digitalis lanata* Ehrh. (foxglove) as a novel source of digoxin: A cardiac glycoside. 3 Biotech..

[6] Cui Y., Yi D., Bai X., Sun B., Zhao Y., Zhang Y. (2012). Ginkgolide B produced endophytic fungus (*Fusarium oxysporum*) isolated from *Ginkgo biloba*. Fitoterapia.

[7] Vigneshwari A., Rakk D., Németh A., Kocsubé S., Kiss N., Csupor D., Papp T., Škrbić B., Vágvölgyi C., Szekeres A. (2019). Host metabolite producing endophytic fungi isolated from *Hypericum perforatum*. PLoS ONE.

[8] Kusari S., Lamshöft M., Spiteller M. (2009). *Aspergillus fumigatus* Fresenius, an endophytic fungus from *Juniperus communis* L. Horstmann as a novel source of the anticancer pro-drug deoxypodophyllotoxin. J. Appl. Microbiol..

[9] Kusari S., Singh S., Jayabaskaran C. (2014). Rethinking production of Taxol^®^ (paclitaxel) using endophyte biotechnology. Trends Biotechnol..

[10] He X., Fang J., Huang L., Wang J., Huang X. (2015). *Sophora flavescens* Ait.: Traditional usage, phytochemistry and pharmacology of an important traditional Chinese medicine. J. Ethnopharmacol..

[11] Chen X., Yi C., Yang X., Wang X. (2004). Liquid chromatography of active principles in *Sophora flavescens* root. J. Chrom. B.

[12] Zhang L., Xu L., Xiao S.-S., Liao Q.-F., Li Q., Liang J., Chen X.-H., Bi K.-S. (2007). Characterization of flavonoids in the extract of *Sophora flavescens* Ait. by high-performance liquid chromatography coupled with diode-array detector and electrospray ionization mass spectrometry. J. Pharma. Biomed. Anal..

[13] Yao Y.Q., Lan F., Qiao Y.M., Wei J.G., Huang R.S., Li L.B. (2017). Endophytic fungi harbored in the root of *Sophora tonkinensis* Gapnep: Diversity and biocontrol potential against phytopathogens. MicrobiologyOpen.

[14] He L., Liu N., Wang Y., Xu H.B., Yu N. (2013). Isolation an antimicrobial action of endophytic fungi from *Sophora flavescens* and effects on microorganism circumstances in soil. Proc. Environ. Sci..

[15] Yu N., He L., Liu N., Wang Y., Xu H., Liu D. (2014). Antimicrobial action of an endophytic fungi from *Sophora flavescens* and structure identification of its active constituent. Biotechnol. Biotechnol. Equip..

[16] Zhang Q., Li Y., Xu F., Zheng M., Xi X., Zhang X., Han C. (2017). Optimization of submerged fermentation medium for matrine production by *Aspergillus terreus*, an endophytic fungus harboring seeds of *Sophora flavescens*, using response surface methodology. Mycobiology.

[17] Saikkonen K., Saari S., Helander M. (2010). Defensive mutualism between plants and endophytic fungi?. Fungal Divers..

[18] Schulz B., Boyle C. (2005). The endophytic continuum. Mycol. Res..

[19] Waller F., Achatz B., Baltruschat H., Fodor J., Becker K., Fischer M., Heier T., Huckelhoven R., Neumann C., von Wettstein D. (2005). The endophytic fungus *Piriformospora indica* reprograms barley to salt-stress tolerance, disease resistance, and higher yield. Proc. Nat. Acad. Sci. USA.

[20] Rodriguez R.J., Woodward C.J., Redman R.S., Southworth D. (2012). Fungal influence on plant tolerance to stress. Biocomplexity of Plant-Fungal Interactions.

[21] Berg G. (2009). Plant–microbe interactions promoting plant growth and health: Perspectives for controlled use of microorganisms in agriculture. Appl. Microbiol. Biotechnol..

[22] Jia M., Chen L., Xin H.-L., Zheng C.-J., Rahman K., Han T., Qin L.-P. (2016). A friendly relationship between endophytic fungi and medicinal plants: A systematic review. Front. Microbiol..

[23] Zhang H.W., Song Y.C., Tan R.X. (2006). Biology and chemistry of endophytes. Nat. Prod. Rep..

[24] Hartley S.E., Gange A.C. (2009). Impacts of plant symbiotic fungi on insect herbivores: Mutualism in a multitrophic context. Annu. Rev. Entomol..

[25] Kajula M., Tejesvi M.V., Kolehmainen S., Mäkinen A., Hokkanen J., Mattila S., Pirttilä A.M. (2010). The siderophore ferricrocin produced by specific foliar endophytic fungi in vitro. Fungal Biol..

[26] Khan A.L., Hamayun M., Kim Y.-H., Kang S.-M., Lee J.-H., Lee I.-J. (2011). Gibberellins producing endophytic *Aspergillus fumigatus* sp. LH02 influenced endogenous phytohormonal levels, isoflavonoids production and plant growth in salinity stress. Proc. Biochem..

[27] Khan A.L., Hamayun M., Kang S.-M., Kim Y.-H., Jung H.-Y., Lee J.-H., Lee I.-J. (2012). Endophytic fungal association via gibberellins and indole acetic acid can improve plant growth under abiotic stress: An example of *Paecilomyces formosus* LHL10. BMC Microbiol..

[28] Khan S., Hamayun M., Yoon H., Kim H.-Y., Suh S.-J., Hwang S.-K., Kim J.-M., Lee I.-J., Choo Y.-S., Yoon U.-H. (2008). Plant growth promotion and *Penicillium citrinum*. BMC Microbiol..

[29] White T.J., Bruns T., Lee S., Taylor J., Innis M.A., Gelfand D.H., Sninsky J.J., White T.J. (1990). Amplification and direct sequencing of fungal ribosomal RNA genes for phylogenetics. PCR Protocols: A Guide to Methods and Applications.

[30] Nath R., Sharma G.D., Barooah M. (2015). Plant growth promoting endophytic fungi isolated from tea (*Camellia sinensis*) shrubs of assam, India. AEER.

[31] Cao Y., Fu D., Liu T., Guo G., Hu Z. (2018). Phosphorus solubilizing and releasing bacteria screening from the rhizosphere in a natural wetland. Water.

[32] Niewolak S. (1980). Occurrence of microorganisms in fertilized lakes. II. Lecithin-mineralizing microorganisms. Pol. Arch. Hydrobiol..

[33] Schwyn B., Neilands J.B. (1987). Universal chemical assay for the detection and determination of siderophores. Anal. Biochem..

[34] Bric J.M., Bostock R.M., Silverstonet S.E. (1991). Rapid in situ assay for indoleacetic acid production by bacteria immobilized on a nitrocellulose membrane. Appl. Environ. Microbiol..

[35] Shah S., Shrestha R., Maharjan S., Selosse M.-A., Pant B. (2018). Isolation and characterization of plant growth-promoting endophytic fungi from the roots of *Dendrobium moniliforme*. Plants.

[36] Horváth E., Brunner S., Bela K., Papdi C., Szabados L., Tari I., Csiszár J. (2015). Exogenous salicylic acid-triggered changes in the glutathione transferases and peroxidases are key factors in the successful salt stress acclimation of *Arabidopsis thaliana*. Funct. Plant. Biol..

[37] Marik T., Tyagi C., Balázs D., Urbán P., Szepesi Á., Bakacsy L., Endre G., Rakk D., Szekeres A., Andersson M.A. (2019). Structural diversity and bioactivities of peptaibol compounds from the Longibrachiatum clade of the filamentous fungal genus *Trichoderma*. Front. Microbiol..

[38] Faragó D., Sass L., Valkai I., Andrási N., Szabados L. (2018). Plant size offers an affordable, non-destructive method to measure plant size and color in vitro. Front. Plant. Sci..

[39] Gordon S.A., Weber R.P. (1951). Colorimetric estimation of indoleacetic acid. Plant. Physiol..

[40] Rahman A., Bannigan A., Sulaman W., Pechter P., Blancaflor E.B., Baskin T.I. (2007). Auxin, actin and growth of the *Arabidopsis thaliana* primary root: Auxin and actin interaction. Plant. J..

[41] Jimtha J.C., Smitha P.V., Anisha C., Deepthi T., Meekha G., Radhakrishnan E.K., Gayatri G.P., Remakanthan A. (2014). Isolation of endophytic bacteria from embryogenic suspension culture of banana and assessment of their plant growth promoting properties. Plant. Cell Tiss. Organ. Cult..

[42] Waqas M., Khan A.L., Kamran M., Hamayun M., Kang S.-M., Kim Y.-H., Lee I.-J. (2012). Endophytic fungi produce gibberellins and indoleacetic acid and promotes host-plant growth during stress. Molecules.

[43] Khan A.L., Al-Harrasi A., Al-Rawahi A., Al-Farsi Z., Al-Mamari A., Waqas M., Asaf S., Elyassi A., Mabood F., Shin J.-H. (2016). Endophytic fungi from frankincense tree improves host growth and produces extracellular enzymes and indole acetic acid. PLoS ONE.

[44] Al-Hosni K., Shahzad R., Latif Khan A., Muhammad Imran Q., Al Harrasi A., Al Rawahi A., Asaf S., Kang S.-M., Yun B.-W., Lee I.-J. (2018). *Preussia* sp. BSL-10 producing nitric oxide, gibberellins, and indole acetic acid and improving rice plant growth. J. Plant. Inter..

[45] Ismail, Hamayun M., Hussain A., Iqbal A., Khan S.A., Lee I.-J. (2018). Endophytic fungus *Aspergillus japonicus* mediates host plant growth under normal and heat stress conditions. BioMed Res. Inter..

[46] Numponsak T., Kumla J., Suwannarach N., Matsui K., Lumyong S. (2018). Biosynthetic pathway and optimal conditions for the production of indole-3-acetic acid by an endophytic fungus, *Colletotrichum fructicola* CMU-A109. PLoS ONE.

[47] Dovana F., Mucciarelli M., Mascarello M., Fusconi A. (2015). In vitro morphogenesis of *Arabidopsis* to search for novel endophytic fungi modulating plant growth. PLoS ONE.

[48] Mehmood A., Khan N., Irshad M., Hamayun M., Husna I., Javed A., Hussain A. (2018). IAA producing endopytic fungus *Fusariun oxysporum* wlw colonize maize roots and promoted maize growth under hydroponic condition. Eur. J. Exp. Biol..

